# Hepatic Osteodystrophy—Molecular Mechanisms Proposed to Favor Its Development

**DOI:** 10.3390/ijms20102555

**Published:** 2019-05-24

**Authors:** Sabrina Ehnert, Romina H. Aspera-Werz, Marc Ruoß, Steven Dooley, Jan G. Hengstler, Silvio Nadalin, Borna Relja, Andreas Badke, Andreas K. Nussler

**Affiliations:** 1 Siegfried Weller Research Institute, Department of Trauma and Reconstructive Surgery, Eberhard Karls University Tuebingen, BG Trauma Center Tuebingen, 72076 Tuebingen, Germany; rominaaspera@hotmail.com (R.H.A.-W.); m.ruoss@hotmail.de (M.R.); abadke@bgu-tuebingen.de (A.B.); andreas.nuessler@gmail.com (A.K.N.); 2 Department of Medicine II, Molecular Hepatology, Medical Faculty Mannheim, University of Heidelberg, 68167 Mannheim, Germany; steven.dooley@medma.uni-heidelberg.de; 3 IfADo-Leibniz Research Centre for Working Environment and Human Factors, Technical University Dortmund, 44139 Dortmund, Germany; hengstler@ifado.de; 4 Department of General, Visceral and Transplant Surgery, University Hospital Tuebingen, 72076 Tuebingen, Germany; silvio.nadalin@med.uni-tuebingen.de; 5 Department of Trauma, Hand and Reconstructive Surgery, University Hospital Frankfurt, Goethe University, 60590 Frankfurt, Germany; info@bornarelja.com

**Keywords:** bone metabolism, osteopenia, osteoporosis, liver disease, hepatic osteodystrophy, vitamin D metabolism, transforming growth factor beta (TGF-β), bone morphogenetic proteins (BMPs), histone deacetylases (HDACs), sclerostin

## Abstract

Almost all patients with chronic liver diseases (CLD) show altered bone metabolism. Depending on the etiology, this manifests in a severe osteoporosis in up to 75% of the affected patients. Due to high prevalence, the generic term hepatic osteodystrophy (HOD) evolved, describing altered bone metabolism, decreased bone mineral density, and deterioration of bone structure in patients with CLD. Once developed, HOD is difficult to treat and increases the risk of fragility fractures. Existing fractures affect the quality of life and, more importantly, long-term prognosis of these patients, which presents with increased mortality. Thus, special care is required to support the healing process. However, for early diagnosis (reduce fracture risk) and development of adequate treatment strategies (support healing of existing fractures), it is essential to understand the underlying mechanisms that link disturbed liver function with this bone phenotype. In the present review, we summarize proposed molecular mechanisms favoring the development of HOD and compromising the healing of associated fractures, including alterations in vitamin D metabolism and action, disbalances in transforming growth factor beta (TGF-β) and bone morphogenetic protein (BMP) signaling with histone deacetylases (HDACs) as secondary regulators, as well as alterations in the receptor activator of nuclear factor kappa B ligand (RANKL)–osteoprotegerin (OPG) system mediated by sclerostin. Based on these mechanisms, we give an overview on the limitations of early diagnosis of HOD with established serum markers.

## 1. Hepatic Osteodystrophy—Definition and Prevalence

Current studies show that almost 75% of patients with chronic liver diseases (CLD) sooner or later suffer from severe osteoporosis [1,2]. Based on this high prevalence, the generic term hepatic osteodystrophy (HOD) evolved, defining alterations in bone mineral metabolism in patients with CLDs [3], eventually resulting in reduced bone mineral densities (BMD) and deterioration of bone structure, e.g., trabecular architecture or bone geometry. These alterations in bone structure increase the risk of fragility fractures in patients with HOD [4,5,6,7,8]. In case of a fracture, reconstruction of bone and handling of surrounding soft tissue represents a great challenge. Poor bone quality complicates classical fixation of fractures with screws and implants; despite continuous development of new products (screws, plates and implants, bone cements, etc.), the rate of delayed healings and non-unions remains high in these patients. Complicated wound healing and altered immune responses additionally raise the risk of infections. The resulting delay in convalescence affects not only quality of life, but also long-term prognosis of patients with CLD due to an increased mortality [8,9].

The most widely studied group describes bone metabolic changes during viral liver diseases (hepatitis B virus (HBV) and hepatitis C virus (HCV)), which strongly depend on the reported disease stage. While on average 37.9% of patients with chronic viral hepatitis show changes in bone mineral metabolism, 80.3% of patients with viral cirrhosis develop a severe osteoporosis [10,11,12]. Metabolic bone disease associated with cholestatic liver diseases is less frequently reported. With an overall rate of 32.4% of patients with primary biliary cirrhosis (PBC) and 42.3% of patients with primary sclerosing cholangitis (PSC) being affected, this group has a high prevalence of developing an osteopenia or osteoporosis [1,13,14,15,16,17,18]. With alcoholism being an independent factor for the manifestation of an osteoporosis, overall 35.9% of patients suffering from alcoholic liver disease show altered bone metabolism and structure [12,19,20,21]. Less is known about non-alcoholic fatty liver disease (NAFLD) or non-alcoholic steatohepatitis (NASH). The mean prevalence of HOD in this patient group, often composed of children, is reported to be 45.7% [22,23,24]. Approximately every second patient (49.3%) with hemochromatosis shows altered bone structure (osteopenia or osteoporosis) [25,26,27]. The same holds for patients with Wilson disease, which have an average HOD rate of 49.3% [28,29,30,31]. The described alterations in BMD significantly increase the risk and cumulative incidence of fractures in these patients, as reported by Tsai et al. investigating almost 4000 cirrhotic patients of mixed etiologies [6]. Table 1 gives an overview of available studies on HOD.

Regardless of the etiology, prevalence and severity (osteopenia or osteoporosis) of HOD positively correlates with duration and severity of the liver disease. Especially during end-stage liver disease and directly after orthotopic liver transplantation (OLT), higher fracture rates are reported, as the need for immunosuppressive drugs, e.g., glucocorticoids, may additionally harm the bone [48]. However, after successful OLT, when the use of glucocorticoids is reduced and liver function is reestablished, BMD frequently recovers [49,50,51,52].

## 2. Limitations of Current Diagnostic Tools for HOD

Although deleterious effects of CLD on bone metabolism and structure are frequently reported, early diagnosis of an altered bone metabolism represents a huge challenge. Radiologic changes in bone, preferably detected by dual-energy X-ray absorptiometry [53,54], often manifest only when bone metabolism is affected over a longer period of time and when changes in BMD, e.g., osteopenia and osteoporosis, are manifested. However, at the point when BMD is decreased, fracture risk is already increased [53,54]. Therefore, it is desirable to identify bone metabolic changes as early as possible in order to prevent or delay loss in BMD.

**Figure 1:**
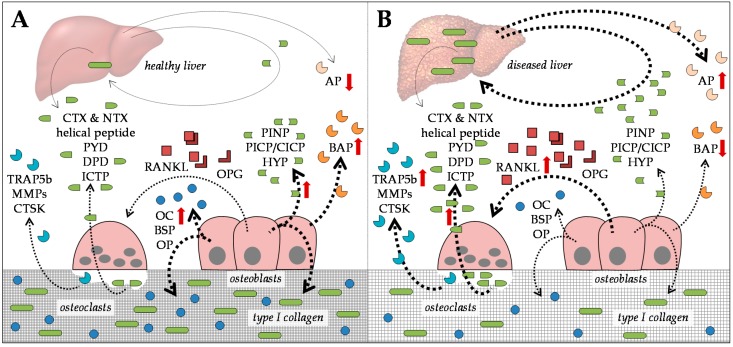
Established serum markers for bone turnover in the context of (**A**) healthy liver and (**B**) diseased liver. Bone resorption markers: tartrate-resistant acid phosphatase isoform 5b (TRAP5b), matrix metalloproteinase isoforms 2, 9, 13, and 14 (MMPs), cathepsin K (CTSK), pyridinolin (PYD), desoxypyridinolin (DPD), helical peptide, type I collagen cross-linked C-telopeptide (ICTP), and C- and N-telopeptide crosslinks of type I collagen (CTX and NTX). Regulators of osteoclastogenesis: receptor activator of nuclear factor kappa B ligand (RANKL) and osteoprotegerin (OPG). Bone formation markers: osteocalcin (OC) bone sialoprotein (BSP), osteopontin (OP), bone-specific alkaline phosphatase (BAP), hydroxyprolin (HYP), and type I collagen N- and C-terminal propeptides (PINP and PICP/CICP). Marker for liver/tissue damage: alkaline phosphatase (AP). Dotted arrows indicate expression. Red arrows indicate altered expression (up or down) in CLD.

Osteoblasts and osteoclasts actively secrete factors into the blood. The detection of these serum markers is established as a marker for bone metabolism. In theory, detection of these serum markers could help identify changes in bone metabolism prior to manifestation of osteoporosis. However, in the context of CLD, the established serum markers for bone turnover allow only limited conclusions, as they often demonstrate the production and degradation of collagen (Figure 1).

### 2.1. Serum Markers for Bone Formation

Characteristic for collagen-I production are increased levels of hydroxyprolin (HYP), and type I collagen N- and C-terminal propeptides (PINP and PICP/CICP) in serum [55,56,57,58]. These markers are frequently used to assess bone formation. However, extensive matrix formation in the diseased liver is also known to increase serum levels of these markers and, thus, lead to false-positive results. Alternative markers for bone formation are increased serum levels of proteins secreted by osteoblasts. Available assays are bone-specific alkaline phosphatase (BAP) and osteocalcin (OC). Quantitative detection methods (ELISA, enzyme immunoassay (EIA), or radioimmunoassay (RIA)) for other proteins secreted by osteoblasts, e.g., bone sialoprotein (BSP), osteopontin (OP), receptor activator of nuclear factor kappa B ligand (RANKL), or osteoprotegerin (OPG), are available for experimental use, but still require validation for use in routine diagnostics. Furthermore, it remains to be elucidated whether these markers are affected by CLD. Taking, for example, alkaline phosphatase (AP), if not distinguished between the specific isoforms, it can be upregulated in patients’ serum as a response to liver/tissue damage (AP) or during bone formation (BAP).

### 2.2. Serum Markers for Bone Degradation

Degradation of collagen-I is accompanied by increased levels of pyridinolin (PYD), desoxypyridinolin (DPD), helical peptide, its cross-linked C-telopeptide (ICTP), and its C- and N-telopeptide crosslinks (CTXα, CTXβ, and NTX), all either circulating in the blood or secreted into the urine [55,56,57,58]. However, not only is excessive collagen formation characteristic during the development of fibrotic and cirrhotic liver diseases, but so is its remodeling. Therefore, excessive matrix degradation in the bone can easily be masked by the diseased liver [59], which limits the use of these markers. Alternative markers for bone resorption are increased serum levels of proteins secreted by osteoclasts; a validated assay is available for the quantification of tartrate-resistant acid phosphatase isoform 5b (TRAP5b) serum levels. Similar to markers representing osteoblast function, quantitative detection methods for other markers of osteoclast function, e.g., cathepsin K (CTSK) or matrix metalloproteinase (MMP) isoforms 2, 9, 13, and 14, are available for experimental use, with the same restrictions; in addition to the lack of validation, it is likely that these markers are affected by CLD, e.g., as reported for MMPs [60,61].

The described limitations are one reason why HOD is often diagnosed only when changes in BMD became manifest in osteopenia or osteoporosis and the affected patients experience fragility fractures. Once developed, HOD is difficult to treat, and special care is required to support healing of existing fractures, as, in these patients, the process is commonly delayed and rich in complications, which in turn negatively affects the etiopathology of the associated liver disease [9]. For early diagnosis and to develop adequate treatment strategies, it is essential to understand the underlying mechanisms leading to HOD.

## 3. Common Risk Factors Favoring the Development of HOD

Several factors are associated with HOD, which are, thus, classified as possible risk factors for disease development. These factors include, among others, age, body mass index (BMI), duration and severity of the underlying liver disease, malnutrition or dietary deficiencies, overall low BMD with a history of fragility fractures, genetic predisposition, hormonal status, iron and copper accumulation, hyperbilirubinemia, alterations in vitamin status, and the effects of the used medication. Table 2 gives an overview on risk factors and proposed underlying mechanisms.

These mainly anamnestic factors define the risk of a patient with CLD to develop severe osteoporosis, but will not give any information on the actual disease status or the onset of the HOD. Furthermore, these factors provide only limited information on underlying molecular mechanisms, required for the identification of therapeutic targets and the development of treatment strategies.

Several of these factors, e.g., age, duration and severity of the underlying CLD, genetic predisposition, decreased BMD, or a familial history of fragility fractures, cannot be affected by treatment. Lifestyle-associated factors, e.g., physical exercise, dietary deficiencies, and consumption of alcohol and cigarettes, however, can be actively influenced by the patients. Thus, patients with CLD should be encouraged to change their lifestyle in order to reduce the risk and delay the development of a HOD. Factors involving medication, and altered hormonal or vitamin status can be influenced with the help of the attending physician. However, improved prevention will require a better understanding on the underlying molecular mechanisms.

## 4. Alterations in Vitamin D and Calcium in Patients with CLD

### 4.1. Vitamin D Metabolism in Patients with CLD

Lipophilic vitamin D (VitD) exists in two natural variants: VitD_2_ (ergocalciferol—in plants) and VitD_3_ (cholecalciferol—in animals). In the human body, VitD is available mainly from cutaneous synthesis (VitD_3_) and to a lesser extent from dietary uptake (VitD_2_ and VitD_3_) [108]. During cutaneous synthesis, 7-dehydrocholesterol (7-DHC) converts to pre-VitD_3_ and VitD_3_ under ultraviolet B (UVB) irradiation [109]. In the liver, 7-DHC is not only synthesized from cholesterol by cholesterol 7α-hydroxylase (CYP7A1), but also degraded to cholesterol by 7-dehydrocholesterol reductase (DHCR7). Mouse models for HOD show increased expression of DHCR7 in the diseased livers, which causes increased 7-DCH degradation during CLD [110,111]. This finding was confirmed by analysis of cirrhotic liver tissue of patients [110].

For circulation in the blood, VitD and its metabolites have to bind vitamin-D-binding protein GC (DBP), which is expressed in the liver. In mice, DBP expression decreases with progression of CLD [110,111]. Thus, it is assumed that circulation of VitD and its metabolites is impaired in patients with advanced liver disease.

**Figure 2:**
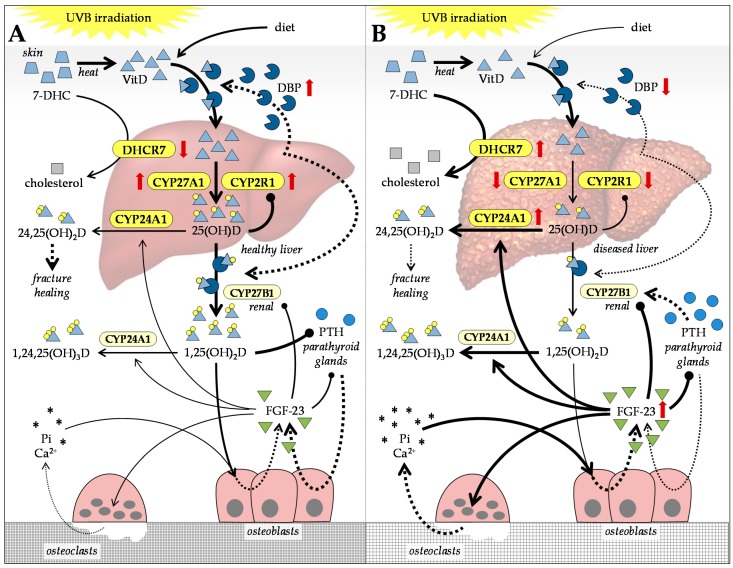
Vitamin D (VitD) metabolism in the context of (**A**) healthy liver and (**B**) diseased liver. In the presence of ultraviolet B (UVB) irradiation and heat, 7-dehydrocholesterol (7-DHC) is processed to VitD in the skin. VitD is sequentially hydroxylated in the liver and the kidneys to its metabolites calcidiol (25(OH)D), calcitriol (1,25(OH)D), 24,25-dihydroxyvitamin D (24,25(OH)D), and 1,24,25-trihydroxy-vitamin D (1,24,25(OH)D). Enzymes involved in VitD metabolism: 7-dehydrocholesterol reductase (DHCR7), vitamin D 25-hydroxylase (CYP2R1), sterol 27-hydroxylase (CYP27A1), 25-hydroxyvitamin D 1-hydroxylase (CYP27B1), and 25-hydroxyvitamin D 24-hydroxylase (CYP24A1). VitD and its metabolites bind to the vitamin-D-binding protein GC (DBP) for transport in the blood. Other regulators: calcium (Ca^2+^), inorganic phosphate (Pi), fibroblast growth factor 23 (FGF-23), and parathyroid hormone (PTH). Dotted arrows indicate expression. Red arrows indicate altered expression (up or down) in CLD.

In the healthy liver, VitD is hydroxylated by VitD 25-hydroxylase (CYP2R1) and sterol 27-hydroxylase (CYP27A1). Expression of both enzymes is reported to be decreased in fibrotic and cirrhotic livers [110,111]. Zhao et al. showed decreased CYP27A1 levels, but not decreased CYP2R1 levels in patients with liver cirrhosis [79]. The reaction product, calcidiol, also called 25-hydroxyvitamin D (25(OH)D), is reported to be decreased during CLD [79,110,112]. In the kidneys, 25(OH)D is further hydroxylated to the biologically active calcitriol, also called 1,25-dihydroxyvitamin D (1,25(OH)_2_D), by 25-hydroxyvitamin D 1α-hydroxylase (CYP27B1) [113,114]. Both 25(OH)D and 1,25(OH)_2_D may be further hydroxylated by 25-hydroxyvitamin D 24-hydroxylase (CYP24A1) in order to facilitate excretion of the products 24,25-dihydroxyvitamin D (24,25(OH)_2_D) and 1,24,25-trihydroxyvitamin D (1,24,25(OH)_3_D). Zhao et al. reported increased CYP24A1 levels in patients with liver cirrhosis, suggesting not only decreased VitD activation and circulation, but also increased VitD degradation in these patients [79]. For an overview, see Figure 2.

### 4.2. VitD-Dependent Cellular Effects Affected in Patients with CLD

To induce effects in target cells, 1,25(OH)_2_D needs to bind to the vitamin D receptor (VDR), which forms heterodimers with related receptors (e.g., the retinoid X receptor) in order to activate intracellular signaling cascades and to bind to vitamin D response elements (VDREs) [115,116]. So far, little is known about effects of CLD on the cellular sensitivity toward 1,25(OH)_2_D, e.g., by regulating expression of VDR and related receptors, components that are strongly regulated by genetic polymorphisms [97,98,99,100,101]. In healthy subjects, 1,25(OH)_2_D supports intestinal absorption of calcium and phosphate [77]. In patients with CLD, lowered 1,25(OH)_2_D serum levels may, thus, impede intestinal absorption of inorganic phosphate (Pi) and calcium and, consequently, induce their release from bone matrix, favoring the loss of mineralized bone matrix [76].

Other cells highly responsive to 1,25(OH)_2_D are dendritic and monocytic cells [115]. Reduced activation of these cells may increase the susceptibility toward infections, as observed in the CALCITOP-study which shows an increased rate of wound infections in patients with low 1,25(OH)_2_D (but not 25(OH)D) serum levels [117].

In bone-forming osteoblasts, 1,25(OH)_2_D enhances the expression of RANKL [118]. Upon binding to receptor activator of nuclear factor kappa B (RANK) on immune cells, RANKL induces their differentiation into bone-resorbing osteoclasts [114,119]. Nevertheless, in vivo, VitD and its metabolites inhibit osteoclastogenesis and, thus, are successfully used as supportive drugs to treat osteoporosis [120]. 

### 4.3. Balancing VitD Levels in Patients with CLD

First attempts were done to balance the described VitD deficiencies in order to improve the bone quality in patients with HOD. At first, oral VitD (VitD_3_ or VitD_2_) supplementation appeared to improve the bone quality in mice [121] and seemed to delay the development of HOD in patients [122]. However, more recent studies showed no significant improvement of BMD in HOD despite improved 25(OH)D serum levels [123,124]. Based on the described alterations in VitD metabolism, the question raises if it is sufficient to supplement VitD or if supplementation of its metabolites is required. There is evidence that supplementation of 25(OH)D or 1,25(OH)_2_D is more efficient than supplementation of VitD [125]. Considering possible deficiencies in intestinal absorption and blood transport of VitD in patients with CLD, the route of application also has to be considered. In this case, it is advisable to first screen for serum levels of VitD and its metabolites to identify possible deficiencies [126,127].

Only a combination of oral VitD and bisphosphonates improved BMD in patients with CLD [128,129,130,131], suggesting that VitD supplementation alone is not sufficient to prevent or delay loss of BMD in these patients. Indeed, bisphosphonates represent the primary medical intervention to prevent bone loss in patients with CLD. However, as the review of Danford et al. framed, a benefit of this treatment in terms of fracture reduction remains to be shown in patients with CLD [132]. Therefore, current studies focus more on a better understanding of the molecular mechanisms that trigger the alterations in VitD and the linked calcium metabolism observed in patients with CLD.

### 4.4. Feedback Mechanisms Regulating VitD Levels in Patients with CLD

VitD metabolism is self-regulated through negative feedback mechanisms including calcium and Pi serum levels, fibroblast growth factor 23 (FGF-23), and parathyroid hormone (PTH) [119,133]. Increased 1,25(OH)_2_D and Pi serum levels may increase expression of FGF-23 in osteoblasts, which inhibits expression of PTH in the parathyroid glands [134,135]. It is proposed that binding of 1,25(OH)_2_D to VDR on the parathyroid glands can also directly decrease expression of PTH [136]. On the one hand, increased PTH levels are thought to induce expression of renal CYP27B1 and, thus, favor formation of 1,25(OH)_2_D [137]. This is one reason why PTH and PTH-related peptide analogs may be used as bone anabolic drugs [138,139]. For example, in an experimental model of biliary cirrhosis, administration of PTH 1-34 analog (teriparatide) was able prevent loss of bone mass and structure [140]. 

Reduced PTH levels on the other hand may increase FGF-23 expression in bone cells [134], which stimulates bone turnover by enhancing VitD metabolism both positively and negatively via inhibition of CYP27B1 and induction of CYP24A1 [97]. FGF-23 serum levels are reported to be increased in patients with CLD [141,142,143]. This may explain why increased PTH levels are negatively associated with secondary osteoporosis in these patients [12,143,144] (Figure 2).

In addition to these described mechanisms, 1,25(OH)_2_D levels can be affected by estrogen, glucocorticoids, or calcitonin, which regulate expression of PTH either directly or indirectly. It was reported that estrogen-dependent regulation of PTH requires action of FGF-23 [145]. Glucocorticoids and calcitonin may increase PTH expression by lowering calcium serum levels. Glucocorticoid therapy may induce intestinal malabsorption and impaired renal re-absorption of calcium [146]. Calcitonin, primarily known as a pharmacologic inhibitor of bone resorption, lowers calcium levels by increasing its renal excretion [147,148]. However, in the same line of evidence, it was observed that decreased calcitonin levels, as observed in patients following thyroidectomy, are associated with decreased BMD [149,150,151]. In contrast to these 1,25(OH)_2_D-dependent mechanisms, proteoglycan 4 may directly induce PTH expression [152].

Noteworthy, the non-classical actions of VitD, e.g., regulation of the renin–angiotensin system, may also play a relevant role in mortality and morbidity of patients with secondary osteoporosis [153,154]. This cascade leads to a sequential activation of angiotensin II, which likely has deleterious effects on blood pressure and the vasculature. Thus, decreased levels of 25(OH)D and 1,25(OH)_2_D are thought to predict hepatic and renal decompensation in these patients [155,156].

## 5. Alterations in Transforming Growth Factor-β Superfamily in Patients with CLD

### 5.1. Regulation of Extracellular Matrix Proteins by Members of the Transforming Growth Factor-β Superfamily

In contrast to the progressively decreasing 25(OH)D and 1,25(OH)_2_D levels, CLD causes a permanent increase in active transforming growth factor-β (TGF-β) [157]. Its expression is induced in the context of the fibrogenic response in the liver. By activating hepatic stellate cells and inducing extracellular matrix (ECM) production, TGF-β triggers fibrotic alterations in the liver in CLD of many etiologies [158]. The active TGF-β is then distributed in the entire body via the blood stream, which may affect bone metabolism and fracture healing.

In healthy subjects, TGF-β is by far the most abundant cytokine in bone [159]. TGF-β is secreted in its latent form by osteoblasts and osteoclasts. Upon secretion, the latent TGF-β is incorporated into the bone matrix [160,161]. During bone resorption or fracture, osteoclasts activate TGF-β in their resorption lacuna via proteolytic and acidic hydrolysis [162,163]. The released active TGF-β in bone then functions as a chemoattractant and growth factor for mesenchymal stem/stromal cells (MSCs) and osteoprogenitor cells, which express a large variety of high affinity TGF-β family receptors. Of the three TGF-β isoforms (TGF-β_1–3_), TGF-β_1_ has the strongest chemotactic effect toward cells of the osteoblastic lineage in human. It is thought to regulate not only migration and proliferation, but also to induce expression of ECM genes, e.g., collagen, fibronectin, and the associated integrin receptors in these cells [161,164,165,166,167]. Less is known about TGF-β_2_, which, upon over-expression in mice bones, stimulates bone metabolism, eventually causing an osteoporotic phenotype [168]. TGF-β_3_ is thought to induce MSC differentiation toward the chondrogenic lineage, an essential step in endochondral ossification as observed in the developing skeleton or in the fracture callus during long bone repair [169]. Research focusing mainly on short-term effects of TGF-β on bone cells undoubtedly shows the importance of TGF-β in the initiation of fracture healing [170]. These local and dose-dependent effects are well described in a dog model, applying TGF-β ectopically in order to support mechanical fixation, bone ingrowth, and gap bone formation of unloaded implants [171]. However, patients with CLD frequently have chronically elevated TGF-β levels. This may disguise the described positive effects of TGF-β in bone, which require tightly regulated local gradients of the cytokine.

**Figure 3:**
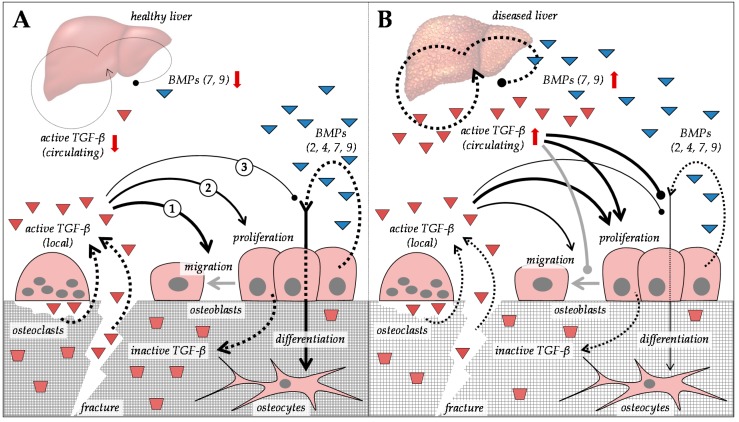
Effects of transforming growth factor-β (TGF-β) and bone morphogenetic protein (BMP) on bone in the context of (**A**) healthy liver and (**B**) diseased liver. Dotted arrows indicate expression. Red arrows indicate altered expression (up or down) in CLD.

Chronically increased levels of active TGF-β, as observed during CLD, alter the composition of the ECM matrix, thus affecting bone flexibility [172,173]. By shifting the ECM matrix toward fibronectin, release of cytokines (including TGF-β) from the bone matrix is induced, which in turn favors osteoclast formation and activity [164,174]. TGF-β signaling induces expression of native fibronectin and its splice variant, termed oncofetal fibronectin in CLD [175,176,177]. This *O*-glycosylated form of fibronectin directly interferes with bone formation and, thus, may contribute to the development of HOD [176]. Similarly, TGF-β induces expression of vimentin via the activating transcription factor ATF4, which may suppress maturation of osteoprogenitor cells and related osteocalcin expression [172] and, thus, contribute to the development of HOD. In addition, chronically elevated TGF-β_1_ levels block osteoblast maturation by interfering with bone morphogenetic protein (BMP) signaling [178]. From the members of the BMP family, BMP2, 4, 7, and 9 show osteo-inductive properties. Interestingly, BMP7 and 9 are expressed in liver cells as a response to damage, initially to suppress pro-fibrotic TGF-β effects [90,91]. While BMPs may suppress TGF-β effects in the liver [91,179], the contrary is the case in bone [178]. Therefore, increased levels of BMPs may not compensate for the inhibitory effects of TGF-β in bone (Figure 3).

### 5.2. Regulation of TGF-β and BMP Signaling

TGF-β and BMP both transduce their signals by binding (usually as homodimers) to a tetrameric receptor complex on the cell surface, consisting of two types of serine/threonine kinase receptors [180,181]. In human, seven type I receptors (termed activin receptor-like kinase (Alk)-1 through 7) and five type II receptors were identified, which have to comply with more than 30 ligands of the TGF-β superfamily. This implies that individual receptors have to bind more than one ligand [182]. Furthermore, cell-type-dependent expression patterns of the receptors may explain the above-described cell-type-dependent differences in ligand response. Upon ligand binding, intracellular signal transduction occurs both canonically (dependent on Smad transcription factors) and non-canonically (Smad-independent/mitogen-activated protein kinase (MAPK) signaling). In bone cells, canonical TGF-β signaling is mediated via Alk4-, 5-, or 7-dependent phosphorylation of Smad2/3, while canonical BMP (BMP2, 4, 7, and 9) signaling is mediated via Alk1-, 2-, 3-, or 6-dependent phosphorylation of Smad1/5/8. Complex formation with Smad4 allows the activated transcription factor complexes to translocate into the nucleus and, thus, regulate target gene expression [183]. Canonical signaling cascades are controlled by various regulatory mechanisms, including among others inhibition by intra- and extracellular inhibitors, regulation of gene expression, post-transcriptional modifications, and intracellular trafficking [184]. Expression of some of the regulatory proteins is initiated by the signaling itself, as an internal feedback mechanism, e.g., the inhibitory Smad6 and 7, Smad ubiquitination regulatory factors (Smurfs)-1 and 2, Smad anchor for receptor activation (SARA), BMP and activin receptor membrane bound inhibitor (BAMBI), Noggin, v-ski sarcoma viral oncogene homolog (Ski), and Ski-like oncogene (SnoN) [178]. Membrane bound BAMBI and soluble Noggin inhibit TGF-β/BMP signaling by competing with the type I receptor for ligand binding [181,185]. The Smad co-factor SARA enhances TGF-β signaling via direct interaction with Smad2, favoring its recruitment to the TGF-β receptor [181]. Intracellularly, Smad6 specifically interferes with the Smad1/5/8 pathway, while Smad7 is able to blunt both Smad1/5/8- and Smad2/3-mediated signal transduction. Mechanistically, inhibitory Smads interact with TGF-β receptors and Smad proteins in order to facilitate their ubiquitination and degradation with the help of the E3 ubiquitin ligases Smurf-1 and 2 [181]. Ski and SnoN belong to the negative regulators of Smad transcriptional function, antagonizing TGF-β signaling primarily through transcriptional modulation via recruitment of nuclear transcriptional co-repressors and histone deacetylases (HDACs) [181].

### 5.3. HDACs as Possible Secondary Regulators for HOD

As described above, disbalanced TGF-β and BMP signaling may favor development of HOD. One proposed mechanism is via recruitment and activation of HDACs [178]. Although numerous HDACs are expressed and active in bone cells, their tight regulation is critical, as different HDACs exert variant effects on the different cell types (Table 3). For example, expression of HDACs 2, 4, 5, 6, and 7 is normally increased during osteogenesis [186,187,188,189,190,191], and deletion/inhibition of HDACs 2, 3, 4, 7, and 5/9 is associated with decreased BMD or altered bone structure [192,193,194,195,196,197,198,199,200,201,202,203,204]. A genome-wide association study even identified HDAC5 as one of 20 loci associated with osteoporosis [205], representing an independent risk factor for the development of HOD. These reports give evidence that HDACs are crucial regulators of bone metabolism. The most commonly described mechanism how HDACs (1, 2, 3, 4, 5, 6, 7, and 8) regulate bone metabolism is their direct interaction or interference with transcription factors involved in osteogenic differentiation, e.g., Runx2, p300, Mef2, Mef2c, NFATc1, Zfp521, or TCF. This interaction usually represses their transcriptional activity and, thus, decreases expression of osteogenic marker genes, e.g., collagen or osteocalcin [187,190,191,206,207,208,209,210,211,212,213,214,215]. In addition to direct interaction with Runx2, HDACs 4 and 5 may further decrease transcriptional activity of Runx2 via post-translational modifications, which favors its degradation [187,210]. Resulting altered expression of target genes, e.g., OPG, RANKL, or Wnt, may affect terminal differentiation of bone cells.

Expression of HDACs is strongly regulated by factors circulating in the blood of patients with CLD. For example, in differentiating human osteoblasts, TGF-β induces expression of HDACs 1, 2, 3, 6, and 11 and blocks expression of HDAC9 [188,189]. On the one hand, a TGF-β-dependent decrease in HDAC9 keeps MSCs and osteoblasts in a proliferative stage, thus preventing their maturation [189]. TGF-β-dependent increase in HDAC6 activity is associated with structural deterioration of primary cilia, the mechanosensors of osteoblasts [188]. Thus, increased active TGF-β levels in the blood of patients with CLD may affect the response of osteoblasts toward mechanical stimulation. Altering primary cilia structure not only affects sensing of mechanical stimuli, but also alters signal transduction cascades, including Ca^2+^, TGF-β, Wnt, and mammalian target of rapamycin (mTOR) [216,217,218]. HDAC6, being induced by hypoxia and oxidative stress [192], may promote transcriptional activity of hypoxia-inducible factor α (Hif-1α) early after fracture [219].

Both HDAC6 and HDAC2 may interact with the glucocorticoid receptor in order to regulate expression of osteogenic genes, e.g., osteocalcin, collagen, or osterix, during osteogenesis, a strongly dose-dependent effect [220,221,222]. This way, increased glucocorticoid signaling, as expected under glucocorticoid medication [102], may contribute to impaired osteogenesis in relevant patients.

Decreased levels of HDACs 3 and 4 are associated with increased bone catabolism, proposedly mediated via increased expression of FGF-21 and MMPs (e.g., MMP3, MMP10, and MMP13) [196,223,224,225]. Interaction with PTH, often increased during CLD, may alter HDAC4-dependent expression of these genes [225,226,227]. Thus, it is feasible that CLD affects bone catabolic effects mediated by HDAC4. By interacting with HDAC5 and Mef2, PTH may increase expression of sclerostin in osteocytes [207], thus preventing formation of mineralized bone matrix.

Diverse small chemical inhibitors for HDAC activity are available; however, their use to support bone metabolism has to be critically discussed, as they were mainly approved for treatment of patients with cancer [250,251,252,253]. Their use is associated with severe adverse effects that may not legitimate their application to delay or prevent the development of HOD. Identification of the actual key regulators (HDAC isoforms) and better understanding of the underlying molecular mechanisms will help choose more specific inhibitors or activators with fewer side effects.

## 6. Sclerostin—A New Player in the Game?

Sclerostin was first identified as relevant for bone formation in studies investigating patients with sclerosteosis and van Buchem disease. In these patients, sclerosing bone conditions are associated with functional loss mutations of the gene (*SOST*) encoding sclerostin [254,255,256].

For a long time, sclerostin was thought to be exclusively expressed in osteocytes as a negative feedback inhibitor to prevent excessive bone formation. Sclerostin was first thought to inhibit BMP-dependent Smad signaling by competing with BMP for receptor binding [257]. However, more recent work revealed that sclerostin, similar to dickkopf 1 (DKK1) and 2 (DKK2), represses Smad1/5/8 signaling indirectly via inhibition of the Wnt signaling pathway, which is required for nuclear translocation of Smad1/5/8 [258].

**Figure 4:**
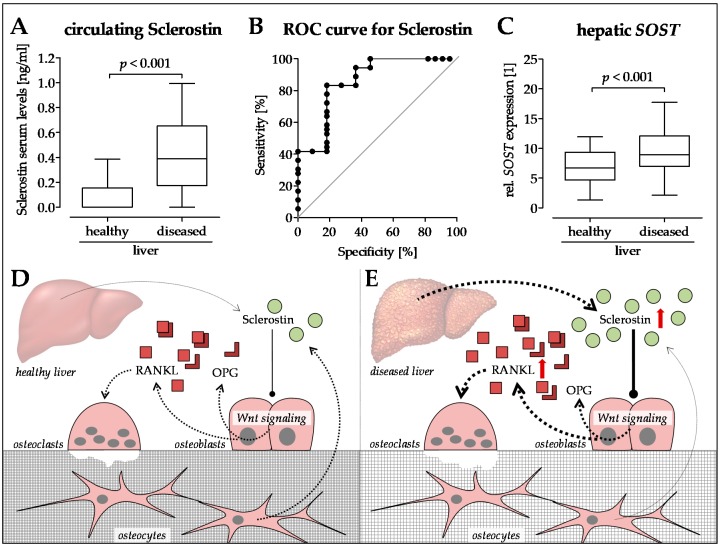
Sclerostin as a possible regulator in the development of hepatic osteodystrophy (HOD). (**A**) Sclerostin serum levels were determined with the help of Sclerostin TECO^®^ ELISA (TECOmedical group, Neufahrn, Germany) in patients with healthy and diseased livers. (**B**) Receiver operating characteristic (ROC) curve with sclerostin as a marker for HOD. (**C**) Expression of *SOST* in healthy and diseased liver tissues. *N* ≥ 22, *n* = 2; statistical comparison with the Mann–Whitney U-test. Proposed regulatory mechanisms in the context of (**D**) healthy and (**E**) diseased liver. Dotted arrows indicate expression. Red arrows indicate altered expression (up or down) in CLD.

Expression of the key regulators of osteoclastogenesis (macrophage colony-stimulating factor (M-CSF), RANKL, and OPG) is regulated by the Wnt signaling cascade. Attenuation of Wnt/BMP signaling by sclerostin affects expression of these genes [259,260]. The resulting imbalances in the RANKL–OPG system may lead to alterations in BMD due to altered activity of osteoclasts. RANKL, by binding to its high-affinity receptor (RANK) located on the surface of monocytes and pre-osteoclasts, drives their differentiation toward mature osteoclasts with bone-resorbing activity [174]. Its secreted inhibitor OPG is produced by the bone and the liver in healthy subjects, in order to inhibit osteoclast differentiation [261]. There are reports showing altered levels of OPG and/or RANKL in patients with CLD [262,263,264]. However, a correlation between decreased BMD in patients with end-stage liver disease was observed only with serum OPG or RANKL levels [265,266] (Figure 4D,E).

In case of increased RANKL levels, therapy with anti-RANKL antibody (Denosumab, Amgen, Thousand Oaks, CA, USA), known under the trade names Prolia and Xgeva, may be an interesting option for these patients [267,268]. Interestingly, in a case report, even amelioration of hepatitis is described when this treatment was applied to a woman with growth hormone deficiency [269].

We identified increased sclerostin serum levels in patients with CLD (liver cirrhosis) (Figure 4A), which is in line with reports on patients with alcoholic and non-alcoholic liver disease [270,271,272]. The work of Guañabens and colleagues suggests that increased sclerostin in diseased liver (PBC) is mainly located in the bile ducts [273]. In our patients, sclerostin serum levels were correlated with decreased BMD (Figure 4B). Thus, treatment with the anti-sclerostin antibody (Romosozumab—AMG 785, UCB, Union Chimique Belge, Brussels, Belgium) may be an interesting strategy to fight osteoporosis in patients with HOD [274,275]. Interestingly, sclerostin was strongly expressed in liver tissues of these patients (Figure 4C). Possible effects on liver disease, however, are yet to be investigated. Circulating via the blood stream, increased sclerostin levels may affect bone metabolism and, thus, contribute to the development of HOD.

## 7. Summary and Outlook

Studies reporting bone changes in patients with CLD suggest that approximately 75% of patients with CLD will develop HOD. Once developed, HOD is difficult to treat [9]. When changes in BMD and bone structure are manifest, patients are at high risk of developing fragility fracture [7]. In case of a fracture, convalescence is prolonged and rich in complications, which in turn negatively affects quality of life and long-term prognosis of these patients [9].

We here demonstrate the challenge toward an early diagnosis of HOD, as many established serum markers for bone turnover are adversely regulated by the diseased liver. We describe how acquisition of anamnestic data may predict the risk of a patient to develop HOD, and how, using this knowledge, patients may delay the development of HOD by modifying lifestyle habits or medication in consultation with the attending physician.

The development of treatment strategies to prevent, delay, or reverse the development of HOD, however, requires deeper understanding of the underlying molecular mechanisms. We summarize current knowledge on potential mechanisms and how they qualify as therapeutic targets. These include alterations in VitD metabolism and action, disbalances in TGF-β and BMP signaling, altered expression and action of HDACs, and sclerostin as a regulator of the RANKL–OPG system. MSCs, osteoblasts, osteocytes, and osteoclasts, via altering their function, all represent interesting targets for the development of therapeutic strategies for patients with HOD. However, the diversity of the described risk factors and possible molecular mechanisms emphasize that HOD is a multifactorial disease that cannot easily be prevented via a simple supplementation of just a single factor, but instead may require a combinatory therapy. 

## Figures and Tables

**Table 1:** Rate of hepatic osteodystrophy (HOD) in chronic liver disease (CLD) of various etiologies. CI—confidence interval.

Author, Year		Patients (*N*)		Rate (%)	95% CI	Forest Plot
		HOD	Total			
**Studies on patients with primary biliary cirrhosis (PBC)**						
**1**	Guañabens et al., 1990 [17]	7	20	35.00	9.07–60.93	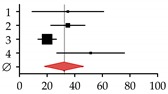
**2**	Lindor et al., 1995 [16]	31	88	35.00	22.64–47.36	
**3**	Menon et al., 2001 [14]	35	176	20.00	13.39–26.61	
**4**	Mounach et al., 2008 [15]	17	33	51.50	27.01–75.99	
**Summary**		90	317	32.35	18.90–45.80	
**Studies on patients with primary sclerosing cholangitis (PSC)**						
**1**	Angulo et al., 1998 [32]	14	81	17.00	8.02–25.98	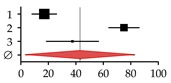
**2**	Angulo et al., 2011 [1]	178	237	75.00	63.97–86.03	
**3**	Keller et al., 2016 [13]	15	40	37.50	18.52–56.48	
**Summary**		207	358	43.18	3.44–82.92	
**Studies on patients with viral hepatitis**						
**1**	Duarte et al., 2001 [33]	25	100	25.00	15.20–34.80	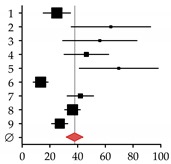
**2**	Auletta et al., 2005 [34]	19	30	64.00	35.37–92.63	
**3**	Hofmann et al., 2008 [35]	17	30	56.00	29.22–82.78	
**4**	Lin et al., 2012 [36]	32	69	46.30	30.24–62.36	
**5**	El-Husseini et al., 2013 [11]	23	33	69.70	41.22–98.18	
**6**	Orsini et al., 2013 [10]	8	60	13.30	4.07–22-53	
**7**	Lai et al., 2015 [37]	25	60	42.00	25.60–58.40	
**8**	Huang et al., 2017 [38]	54	148	36.30	26.59–46.01	
**9**	Bering et al., 2018 [39]	28	104	27.10	17.09–37.11	
**Summary**		231	634	37.87	27.61–48.13	
**Studies on patients with viral cirrhosis**						
**1**	Gallegy-Rojo et al., 1998 [40]	17	32	53.00	27.78–78.22	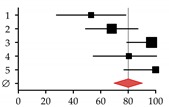
**2**	George et al., 2009 [41]	49	72	68.00	48.95–87.05	
**3**	Choudhary et al., 2011 [12]	112	115	97.00	79.00–115.00	
**4**	Goubraim et al., 2013 [42]	37	46	80.50	54.57–106.43	
**5**	Karoli et al., 2016 [43]	72	72	100.00	76.90–123.10	
**Summary**		287	337	80.27	63.19–97.36	
**Studies on patients with alcoholic liver disease**						
**1**	Spencer et al., 1986 [44]	45	96	46.88	33.18–60.57	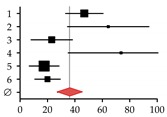
**2**	Diamond et al., 1989 [21]	18	28	64.29	34.59–93-98	
**3**	Gonzalez-Calvin et al., 1993 [45]	9	39	23.08	8.00–38.15	
**4**	Kim et al., 2003 [46]	14	19	73.68	35.09–112.28	
**5**	Malik et al., 2009 [20]	10	57	17.54	6.67–28.42	
**6**	Savic et al., 2014 [19]	6	30	20.00	4.00–36.00	
**Summary**		102	269	37.87	20.61–51.17	
**Studies on patients with non-alcoholic fatty liver disease (NAFDL) or non-alcoholic steatohepatitis (NASH)**						
**1**	Pardee et al., 2012 [22]	17	38	45.00	23.67–66.33	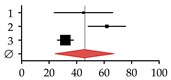
**2**	Kim et al., 2017 [23]	80	129	62.00	48.41–75.59	
**3**	Chen et al., 2018 [24]	116	365	31.80	26.01–37.59	
**Summary**		213	532	45.71	24.50–66.92	
**Studies on patients with hemochromatosis**						
**1**	Diamond et al., 1989 [25]	10	22	45.00	16.97–73.03	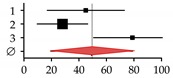
**2**	Sinigaglia et al., 1997 [26]	9	32	28.00	9.67–46.33	
**3**	Guggenbuhl et al., 2005 [27]	30	38	78.90	50.66–107.14	
**Summary**		49	92	49.26	19.41–79.10	
**Studies on patients with Wilson disease**						
**1**	Hegedus et al., 2002 [31]	9	21	43.00	14.95–71.05	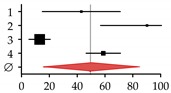
**2**	Selimoglu et al., 2008 [30]	28	31	90.30	56.85–123.75	
**3**	Quemeneur et al., 2014 [29]	11	85	13.00	5.33–20.67	
**4**	Weiss et al., 2015 [28]	87	148	58.80	46.45–71.15	
**Summary**		135	285	49.31	16.10–82.53	

HOD occurrence rates (%) were summarized with the random-effect model described in Neyeloff et al. [47] and visualized with the GraphPad Prism software. The research strategy is summarized in Appendix A.

**Table 2:** Risk factors for bone loss in CLD. BMD—bone mineral densities; OLT—orthotopic liver transplantation; PTH—parathyroid hormone; IGF-1—insulin-like growth factor 1; TNF—tumor necrosis factor; IL-6—interleukin 6.

Risk Factors	Proposed Mechanisms	Ref.
Age	Independent of CLD, age may cause disbalances in osteoclast and osteoblast function. This is often associated with altered hormonal status or epigenetic changes.	[62]
Severity of liver damage	HOD is correlated with severity of the liver disease; HOD is more common in patients with end-stage liver disease and cirrhosis than in patients with fibrosis or hepatitis.	[62]
Low body mass index	A low body mass index (BMI) often correlates with low BMD both in healthy subjects and patients with CLD. A cut-off is usually set at a BMI below 19 kg/m^2^.	[63,64,65]
Dietary deficiencies	Malnutrition or dietary deficiencies frequently occur in patients with CLD (12% of OLT patients), due to altered nutritional requirements during ascites or other complications.	[66,67]
Alcohol consumption	Ethanol affects bone directly via a toxic effect on osteoblasts and indirectly by altering PTH, vitamin D, testosterone, IGF-1, cytokines (e.g., TNF or IL-6) and cortisol levels.	[68,69,70,71]
Cigarette consumption	Independent of CLD, smoking affects osteoblast and osteoclast function, favoring the development of severe osteoporosis and increasing the risk for fragility fractures.	[62]
Physical exercise	In patients with CLD, exercise levels are often reduced compared to healthy individuals; thus, the bone receives less mechanical stimulation.	[72,73]
Muscle wasting	Muscle wasting is very common in patients with CLD. When it occurs independent of malnutrition, it may be an indicator for the manifestation of HOD.	[74]
Hormonal status	Early menopause and post-menopausal status additionally favors bone loss in women.	[64]
	Hypogonadism may cause osteoporosis independent of CLD. Parenchymal damage during CLD may cause hypogonadism due to an altered hypothalamic–pituitary–thyroid function with reduced release of gonadotrophins and primary gonadal failure.	[75]
Anomalies of vitamin D and calcium metabolism	CLD patients may have reduced vitamin D (VitD) absorption in the gut.	[19,76,77,78,79]
	Enterohepatic circulation of VitD might be disturbed in patients with CLD.	
	CLD patients frequently show impaired hepatic hydroxylation of VitD.	
	CLD patients may have increased urinary VitD excretion.	
	Reduced tissue sensitivity to VitD may contribute to the development of HOD.	
	VitD deficiency may cause hyperparathyroidism which increases bone turnover.	[80,81]
Vitamin K deficiency	Vitamin K (VitK) is required for the formation of osteocalcin and osteonectin. VitK inhibits osteoclast viability, maturation, and function.	[82,83,84]
Growth hormones	IGF-1 levels, which decrease during CLD, were linked to HOD.	[85,86]
	CLD is associated with a progressive increase in growth hormone (GH) resistance.	[87]
	Active transforming growth factor β (TGF-β) is produced in inflamed liver tissue.	[88,89]
	In response to damage, liver may produce bone morphogenetic proteins (BMPs).	[90,91]
Iron and copper	Iron may directly affect osteoblast function. An excessive pituitary iron deposition may favor the development of hypogonadism independent of the CLD.	[27,92,93]
Increased bilirubin	Increased levels of unconjugated bilirubin (hyper-bilirubinemia) were associated with a decreased osteoblast function, mediated possibly via regulation of IGF-1.	[66,94,95,96]
Genetic factors	Genetic polymorphisms were described which may favor the development of HOD, including genes encoding vitamin D receptors or collagen type 1A1.	[97,98,99,100,101]
Medication	Corticosteroids affect bone structure by increasing osteoclasts activity and by decreasing differentiation, recruitment, and lifespan of osteoblasts.	[102]
	Calcineurin inhibitors are used in conjunction with corticosteroids; thus, the independent effect of these agents on bone metabolism in humans is uncertain.	[103]
	Antiviral agents, e.g., ribavirin, may directly affect osteoclast and osteoblast function.	[104,105,106]
	Cholestyramine, a bile-acid sequestrant used to treat pruritus or itching during CLD, was reported to adversely affect the intestinal absorption of VitD.	[107]
	The effect of medication, e.g., diuretics, anticoagulants, and chemotherapy, used in the treatment of advanced liver disease, on bone metabolism in humans is uncertain.	

**Table 3:** Proposed regulatory roles of histone deacetylases (HDACs) in bone metabolism. MSC—mesenchymal stem/stromal cells; OPG—osteoprotegerin; FGF-21—fibroblast growth factor 21; MMP—matrix metalloproteinase; Hif-1α—hypoxia-inducible factor α; RANKL—receptor activator of nuclear factor kappa B ligand; PPARγ—peroxisome proliferator-activated receptor γ;.

HDACs		Proposed Mechanisms	References
**Deletion/Inhibition of**	**1, 9**	Expression is decreased during osteogenic differentiation.	[188,189,190,214,228]
	**9**	Expression is blocked by TGF-β signaling.	[188,189]
	**4, 9**	Expression is regulated by microRNAs (miR-17, miR-29b, miR-188).	[229,230,231]
	**1, 6**	Associated with improved skeletal phenotypes.	[228,232,233,234]
	**2–5, 7–9**	Associated with impaired skeletal phenotypes.	[192,193,194,195,196,197,198,199,200,201,202,203,204]
	**1, 7**	Induce expression of osteoblastic genes, e.g., TNAP.	[191,235]
	**3**	Drives MSC differentiation towards adipogenic lineage.	[198,199,200,201]
	**3, 4**	Favor expression of OPG, FGF-21, MMP3, MMP10, and MMP13.	[197,208,223,224,236]
	**4, 5, 9–11**	Increase osteoclast size and demineralization activity, together with increased expression of c-Fos, NFATc1, and Cathepsin K.	[237]
**Increased Expression of**	**2, 4–7**	Increased during osteogenic differentiation.	[186,187,188,189,190,191]
	**1–3, 6, 11**	Induced by TGF-β signaling.	[188,189,190,214,228]
	**1**	Induced by mechanical stimulation.	[228]
	**6**	Induced by hypoxia and oxidative stress.	[192]
	**4, 5**	Associated with impaired skeletal phenotypes.	[186,238]
	**5**	Polymorphisms are associated with decreased BMD.	[205]
	**5, 6**	Suppress expression of transcription factors, e.g., Runx2 or osterix.	[210,220]
	**1–8**	Repress transcriptional activity of, e.g., Runx2, p300, Mef2, Mef2c, NFATc1, Zfp521, or TCF by direct interaction/binding.	[187,190,191,206,207,208,209,210,211,212,213,214,215]
	**4, 5**	Deacetylate Runx2, affecting its transcriptional activity.	[187,210]
	**4, 5**	Deacetylate Runx2, promoting its degradation.	[187,210]
	**4, 9**	Expression is regulated by miRNAs (miR-17, miR-29b, miR-188).	[229,230,231]
	**4**	Its cytoplasmic–nuclear shuttling is regulated by mechanical load.	[194]
	**2**	Regulates proliferation, oxidative stress, and apoptosis by (binding) regulating transcriptional activity of Nrf2/ARE.	[239,240]
	**4**	Interaction with PTH regulates expression of genes, e.g., MMP13.	[225,226,227]
	**6**	Promotes Hif-1α transcriptional activity.	[219]
	**2**	Favors osteoclastogenesis via Akt-mediated suppression of FoxO1.	[241]
	**2, 6**	Interact with glucocorticoid receptor to regulate inflammation and expression of genes, e.g., osteocalcin or collagen during osteogenesis.	[220,221,222,242]
	**3**	Required for bone maintenance during aging.	[243]
	**3**	Represses activity of MMP13, proposed regulation via ERK1/2.	[223,224]
	**5**	Regulates PTH-driven sclerostin expression in osteocytes via Mef2.	[207]
	**6**	Affects structural integrity of primary cilia, the mechanosensory organelle on osteoblasts, which regulates signaling pathways.	[188]
	**3, 7, 9**	Regulate osteoclastogenesis via RANKL, Wnt, and PPARγ.	[244,245,246,247]
	**1, 3, 7**	Regulate inflammation, proposedly involving STAT and NF-κB.	[224,234,248]
	**9**	Promotes proliferation of osteogenic cells, interacting with p53.	[189,249]

## Abbreviations

1,24,25(OH)D	1,24,25-trihydroxyvitamin D
1,25(OH)D	1,25-dihydroxyvitamin D, also called calcitriol
24,25(OH)D	24,25-dihydroxyvitamin D
25(OH)D	25-hydroxyvitamin D, also called calcidiol
7-DHC	7-dehydrocholesterol
BAMBI	BMP and activin receptor membrane bound inhibitor
BAP	bone-specific alkaline phosphatase
BMD	bone mineral density
BMI	body mass index
BMP	bone morphogenetic protein
BSP	bone sialoprotein
CLD	chronic liver disease
CTSK	cathepsin K
CTX	C-telopeptide crosslinks of type I collagen
CYP24A1	25-hydroxyvitamin D 24-hydroxylase
CYP27A1	sterol 27-hydroxylase
CYP27B1	25-hydroxyvitamin D-1α-hydroxylase
CYP2R1	vitamin D-25-hydroxylase
CYP7A1	cholesterol 7α-hydroxylase
DBP	vitamin-D-binding protein GC
DHCR7	7-dehydrocholesterol reductase
DKK1	dickkopf 1
DKK2	dickkopf 2
DPD	desoxypyridinolin
ECM	extracellular matrix
ELISA	enzyme-linked immunosorbent assay
EIA	(solid-phase) enzyme immunoassay
FGF	fibroblast growth factors
GH	growth hormone
HBV	hepatitis B virus
HCV	hepatitis C virus
HDAC	histone deacetylase
HOD	hepatic osteodystrophy
HYP	hydroxyprolin
ICTP	type I collagen cross-linked C-telopeptide
IGF-1	insulin-like growth factor 1
M-CSF	macrophage colony-stimulating factor
MMP	matrix metalloproteinase
MSC	mesenchymal stem/stromal cell
NAFLD	non-alcoholic fatty liver disease
NASH	non-alcoholic steatohepatitis
NTX	N-telopeptide crosslinks of type I collagen
OC	osteocalcin
OLT	orthotopic liver transplantation
OP	osteopontin
OPG	osteoprotegerin
PBC	primary biliary cirrhosis
PDGF	platelet-derived growth factor
Pi	inorganic phosphate
PICP	type I collagen C-terminal propeptide
PINP	type I collagen N-terminal propeptide
PPARγ	peroxisome proliferator-activated receptor γ
PSC	primary sclerosing cholangitis
PTH	parathyroid hormone
PYD	pyridinolin
RANKL	receptor activator of nuclear factor kappa B ligand
RIA	radioimmunoassay
SARA	Smad anchor for receptor activation
Ski	v-ski sarcoma viral oncogene homolog
SnoN	Ski-like oncogene
TGF-β	transforming growth forming factor-β
TRAP5b	tartrate-resistant acid phosphatase isoform 5b
UVB	ultraviolet B
VDR	vitamin D receptor
VRDE	vitamin D response element
VitD	vitamin D
VitD_2_	vitamin D_2_, also called ergocalciferol
VitD_3_	vitamin D_3_, also called cholecalciferol
VitK	vitamin K

## Appendix A

The following search terms were used in PubMed and Web of Science (title and abstract):

“hepatic osteodystrophy”	145 hits
“liver disease” and “bone metabolism”	80 hits
“liver disease” and “osteopenia”	137 hits
“liver disease” and “osteoporosis”	447 hits
“liver disease” and “fractures”	225 hits
“hepatitis” and “bone metabolism”	39 hits
“hepatitis” and “osteopenia”	354 hits
“hepatitis” and “osteoporosis”	340 hits
“hepatitis” and “fractures”	193 hits
“liver fibrosis” or “liver cirrhosis” and “bone metabolism”	48 hits
“liver fibrosis” or “liver cirrhosis” and “osteopenia”	460 hits
“liver fibrosis” or “liver cirrhosis” and “osteoporosis”	375 hits
“liver fibrosis” or “liver cirrhosis” and “fractures”	162 hits

After removal of the duplicates, the manuscripts were screened (abstracts). Only manuscripts available in German and English were considered for further evaluation. Table 1 comprises studies describing human data on patients with defined liver diseases (mixed etiologies are not included).
